# Far lateral approach for resection of lower pontine cavernous malformation

**DOI:** 10.3171/2019.7.FocusVid.19172

**Published:** 2019-07-01

**Authors:** Georgios A. Zenonos, Samir Sur, Maximiliano Nuñez, David T. Fernandes-Cabral, Jacques J. Morcos

**Affiliations:** 1 Department of Neurological Surgery, University of Miami, Miami, Florida; and; 2 Department of Neurological Surgery, University of Pittsburgh Medical Center, Pittsburgh, Pennsylvania

**Keywords:** far lateral approach, brainstem cavernous malformation, brainstem safe entry zone, brainstem anatomy, cavernous malformation, video

## Abstract

In this 3D video we review the case of a lower pontine cavernous malformation in a 31-year-old man who presented with hemiparesis and an abducens palsy. The cavernous malformation was completely resected through a far lateral approach and a peritrigeminal brainstem entry zone, with a significant improvement in the patient’s hemiparesis. The relevant anatomy is reviewed in detail through multiple anatomical brainstem dissection specimens, as well as high-definition fiber tractography images. The rationale for the approach is analyzed relative to other possible options, and a number of technical pearls are provided.

The video can be found here: https://youtu.be/fH2Q7RjlBKQ.

## Transcript

### 0:20 Introduction

In this video we will present the far lateral approach for resection of a lower pontine cavernous malformation. The case refers to a 31 year-old man who presented with a 1-week history of headaches, right arm weakness, and left cranial nerve six palsy. A CT scan revealed a hemorrhagic lesion in the left lower pons, and a T2 MRI showed the classic popcorn appearance of a cavernous malformation. An associated developmental venous anomaly was seen on the contrasted images on the medial border of the lesion. The cavernous malformation presented to the surface of the pons just above the pontomedullary fissure and just superior to the left vertebral artery. Here on the CISS sequence we can see the lesion marked with a star between the exit zone of cranial nerve VI and the VII-VIII complex. It is useful to review some of the brainstem anatomy just above the pontomedullary junction. The motor nuclei of cranial nerves VI and VII are found in the pontine tegmentum, whereas the basis pontis is dominated by the corticospinal tracts. In between the two, we find the spinothalamic tracts straddled by the medial and lateral lemnisci. The hematoma in the basis pontis likely compressed the corticospinal tracts as well as the intraparenchymal course of cranial nerve VI, causing the weakness and opthalmoparesis. A critical component of planning an approach to a brainstem cavernous malformation is the location of the corticospinal tracts. It is useful to remember that the cerebral peduncle projections taper throughout the pons, with mainly corticospinal fibers transitioning to the pyramids at the pontomedullary fissure, as the frontopontine and temporopontine fibers synapse throughout the pons. It is also useful to remember that the exit zone of the abducens nerve is a good approximation for the lateral border of the pyramids. The peritrigeminal entry zone is a well-described entry zone to the ventrolateral pons. It is roughly located between cranial nerves V and VII and courses behind the corticospinal tracts, only disrupting some of the mid and lower transverse pontine fibers. Using the peritrigeminal entry zone there are a few options to approach our lesion. Through a retrosigmoid approach there are typically difficulties in accessing the posterior-most components of the cavernous malformation. The presigmoid retrolabyrinthine approach provides a more ventral angle of attack, and better access to these dorsal components. However, we thought that a far lateral approach would still allow a relatively ventral angle of attack, but would also provide improved access to the pontomedullary fissure, where the lesion presented to the surface, while aligning better with the long axis given its superior extension.

### 2:27 Positioning

The patient was secured in a park bench position and a classic hockey stick incision was planned. The transverse-sigmoid junction is approximated by a point 4.5 cm behind the EAC, on a line connecting the root of the zygoma with the inion. Notably, somatosensory evoked potentials, motor evoked potentials, facial nerve, abducens nerve, lower cranial nerve, and brainstem EMGs were performed throughout the case. Auditory brainstem evoked potentials were also monitored.

### 3:11 Approach

A single myocutaneous flap is elevated leaving a cuff at the superior nuchal line for reapproximation, and a suboccipital craniotomy is performed along with a C1 laminectomy. Some of the paracondylar bone is removed to allow a more ventral angle of attack. The dura is opened in a C-shaped fashion, based laterally, and CSF is released from the cisterna magna, achieving brain relaxation. We proceed with arachnoidal dissection along the accessory nerve. We see the point of entry of the vertebral artery, and the VII-VIII complex as well as the lower cranial nerves are dissected. Cranial nerve V is seen at the depth. Branches of the anterior inferior cerebellar artery are also dissected and preserved. Eventually, the point where the hematoma presents to the surface of the pons is seen just above the pontomedullary fissure. The surface of the pons is stimulated to confirmed the absence of corticospinal tracts. It appeared that a window under the cranial nerves IX and X would provide a better trajectory to the cavernous malformation at the pontomedullary fissure. After the arachnoid adhesions is dissected, a fixed retractor is placed to retract the tonsils and enhance the exposure. AICA branches are dissected and mobilized from the pontomedullary fissure. Eventually the origin of cranial nerve VI comes into view, marking the lateral border of the pyramids and the medial border of our exposure.

### 4:48 Cavernoma resection

After confirming our location and trajectory with image guidance, a small area of the pia is coagulated and opened, and shortly after, the hematoma is encountered. After the liquified hematoma is released, the pial incision is extended a bit, but just within the confines of where the hematoma presents to the surface. A Rhoton No. 6 can be used to bluntly start dissecting the cavernous malformation from the surrounding brainstem parenchyma. In contrast to the dissection technique in other less eloquent areas, the use of bipolar cautery should be limited. Once a plane has been established between the cavernous malformation and the surrounding parenchyma, a variety of instruments can be used to start developing this. These include spreading of the bypass forceps, or the use of a plate dissector from the Rhoton set. Once this plane is further developed and an edge is available, the dusted diamond tip of bypass forceps is a very useful grabbing tool, and the use of traction-countertraction technique with the suction as a dissector can be very useful in further developing this plane. Once a decent amount of dissection has been performed, it is useful to partially debulk the cavernous malformation, so that is easier to work with. The goal should generally be to remove the malformation in as few pieces as possible, so as to maintain a dissection plane and avoid leaving any residual. That being said, removing the malformation in a piecemeal fashion is less traumatic to the surrounding parenchyma and allows for a smaller pial opening. As such, it’s a fine balance and a judgment call to determine when the dissected lesion is becoming too bulky to work with, prompting the surgeons to debulk it by removing a piece. It is important to remember, though, when removing these pieces of the malformation, not to remove too large of a piece and lose the established dissection plane, as this may be difficult to reestablish at the depth of the resection cavity, and when this resection cavity is partially collapsed. The use of pituitary forceps can provide better purchase for larger pieces. A micronerve hook may be very helpful in bringing into view small residual pieces on the side walls of the resection cavity. Again, bipolar cautery should only be used in a very targeted fashion for focal bleeding points. Some final residual pieces are removed from the medial wall of the resection cavity, taking care to avoid injury to the developmental venous anomaly. A careful final inspection of the resection cavity reveals no residual cavernoma. The surrounding hemosiderin ring is not chased in such eloquent location.

### 8:04 Closure and postoperative course and imaging

The dura is closed in a watertight fashion, followed by replacement of the bone flap. The muscle is closed in multiple layers, followed by a running locked Prolene on the skin. Postoperative imaging revealed complete resection of the malformation. The patient had a dramatic improvement in his upper extremity strength, but minimal improvement in his abducens palsy at 2 weeks of follow-up. He had no new neurologic deficits or other complications.
